# Principles of Diagnosis and Treatment in Heart Affections

**Published:** 1916-12

**Authors:** 


**Principles of Diagnosis and Treatment in Heart Affections.** By Sir James Mackenzie, M.D., F.R.S. Pp. viii. 264. Henry Frowde, London. 1916.

The author has prepared former postgraduate lectures so as to make a series of chapters on various heart conditions, many problems of which have been elucidated by means of the polygraph and electro-cardiograph, or other instruments to which Sir James has devoted the most careful attention, and has himself devised or improved. Yet the first chapter suffices to remind the reader that these mechanical aids to research and diagnosis, closely associated as they are with the author's work, are in his estimation but means to an end. Hence much stress is laid on the proper appreciation of the patient's sensations as enabling the physician to understand many obscure complaints ; as, for example, the recognition of abnormal heart action, and the author emphasises the necessity for studying the patient's sensations while they are present, and all associated phenomena carefully noted, and he considers that the general practitioner alone has the real opportunity for properly investigating these phenomena. A good deal of space is devoted to the subjective and objective signs of heart failure. But perhaps the author is at his best when discussing heart irregularities, murmurs, auricular fibrillation, while his section on the principles of treatment is of the utmost value.

